# The emergence and worldwide spread of the species *Trichophyton indotineae* causing difficult-to-treat dermatophytosis: A new challenge in the management of dermatophytosis

**DOI:** 10.1371/journal.ppat.1010795

**Published:** 2022-09-29

**Authors:** Anuradha Chowdhary, Ashutosh Singh, Amtoj Kaur, Ananta Khurana

**Affiliations:** 1 Medical Mycology Unit, Department of Microbiology, Vallabhbhai Patel Chest Institute, University of Delhi, Delhi, India; 2 National Reference Laboratory for Antimicrobial Resistance in Fungal Pathogens, Vallabhbhai Patel Chest Institute, University of Delhi, Delhi, India; 3 Department of Dermatology, Dr. RML Hospital and Atal Bihari Vajpayee Institute of Medical Sciences, New Delhi, India; University of Maryland, Baltimore, UNITED STATES

## Introduction

Superficial fungal infections of the skin, hair, and nails are the fourth most common cause of human disease affecting 20% to 25% of the world’s population [1]. Dermatophytosis is a superficial fungal infection caused by dermatophytic fungi that affect skin and the keratinized structures (hair and nails) arising from it. Dermatophytes, especially from the genera *Trichophyton*, cause the majority of superficial mycoses. These infections result in considerable morbidity and economic burden on the healthcare system [2]. In recent years, an alarming increase in the frequency of recalcitrant superficial fungal infections caused by novel species of *Trichophyton*, i.e., *Trichophyton indotineae* has been witnessed worldwide [3–24]. Importantly, the majority of the *T*. *indotineae* strains exhibit alteration in the squalene epoxidase (*SQLE*) gene that confers high terbinafine (TRB) resistance [3–5,7,10,11,13–17,19,21,22,25–28]. TRB is a first-line drug for treatment of moderate to severe dermatophytosis, and patients with *T*. *indotineae* infections typically show decreased effectiveness of oral therapy with this antifungal [29]. *T*. *indotineae* has been designated recently in the year 2020 as a distinct species independent of *Trichophyton interdigitale* and *Trichophyton mentagrophytes* on the basis of internal transcribed spacer (ITS) region sequencing of 2 highly TRB-resistant *Trichophyton* strains from a Nepali patient and an Indian patient [5]. On ITS phylogenetic analysis, TRB-resistant Indian strains cluster independently of the clusters of the *T*. *interdigitale* and *T*. *mentagrophytes* strains and differ in 2 to 3 single-nucleotide polymorphisms (SNPs) from *T*. *mentagrophytes*/*T*. *interdigitale* strains [5]. Subsequently, multigene polyphasic analysis of a larger data set of *T*. *indotineae* strains showed that these strains have distinct sequences of the high mobility group (HMG) gene as compared to *T*. *mentagrophytes* s. str. and *T*. *interdigitale* s. str [30]. Unlike the infections caused by *T*. *mentagrophytes* and *T*. *interdigitale*, *T*. *indotineae* often presents with extensive skin lesions and a chronic relapsing course. The whole genome sequencing analysis of 20 *T*. *indotineae* strains demonstrate that this new species is distinct clonal offshoot of *T*. *mentagrophytes*/*T*. *interdigitale* spp. complex. Thus, naming of this emerging antifungal-resistant species was essential as it could not be unambiguously identified as either *T*. *mentagrophytes* or *T*. *interdigitale* based on ITS sequencing, mycological and physiological characteristics.

In the last few years, dermatophytosis due to *T*. *indotineae* has not been limited to the Indian subcontinent but has also spread to Europe, Middle East, and North America related to travel and migration [7–24]. Further, reports of increasing treatment failure and acquisition of drug resistance in these difficult to treat *T*. *indotineae* infections have brought this entity to forefront due to limited alternative therapies. Although the significance of this problem has not gained global attention, it is just a matter of time when recalcitrant superficial dermatophytosis will be a potential public health threat worldwide. In this update, we apprise the emergence of *T*. *indotineae* in the Indian subcontinent and its rapid worldwide migration. Further, we highlight the challenges in the mycological identification and impact of the drug resistant *T*. *indotineae* strains on treatment of dermatophytosis.

## A unique terbinafine-resistant *Trichophyton* species, *T*. *indotineae*, is causing alarming, difficult-to-treat dermatophytosis in India

**Fig 1:**
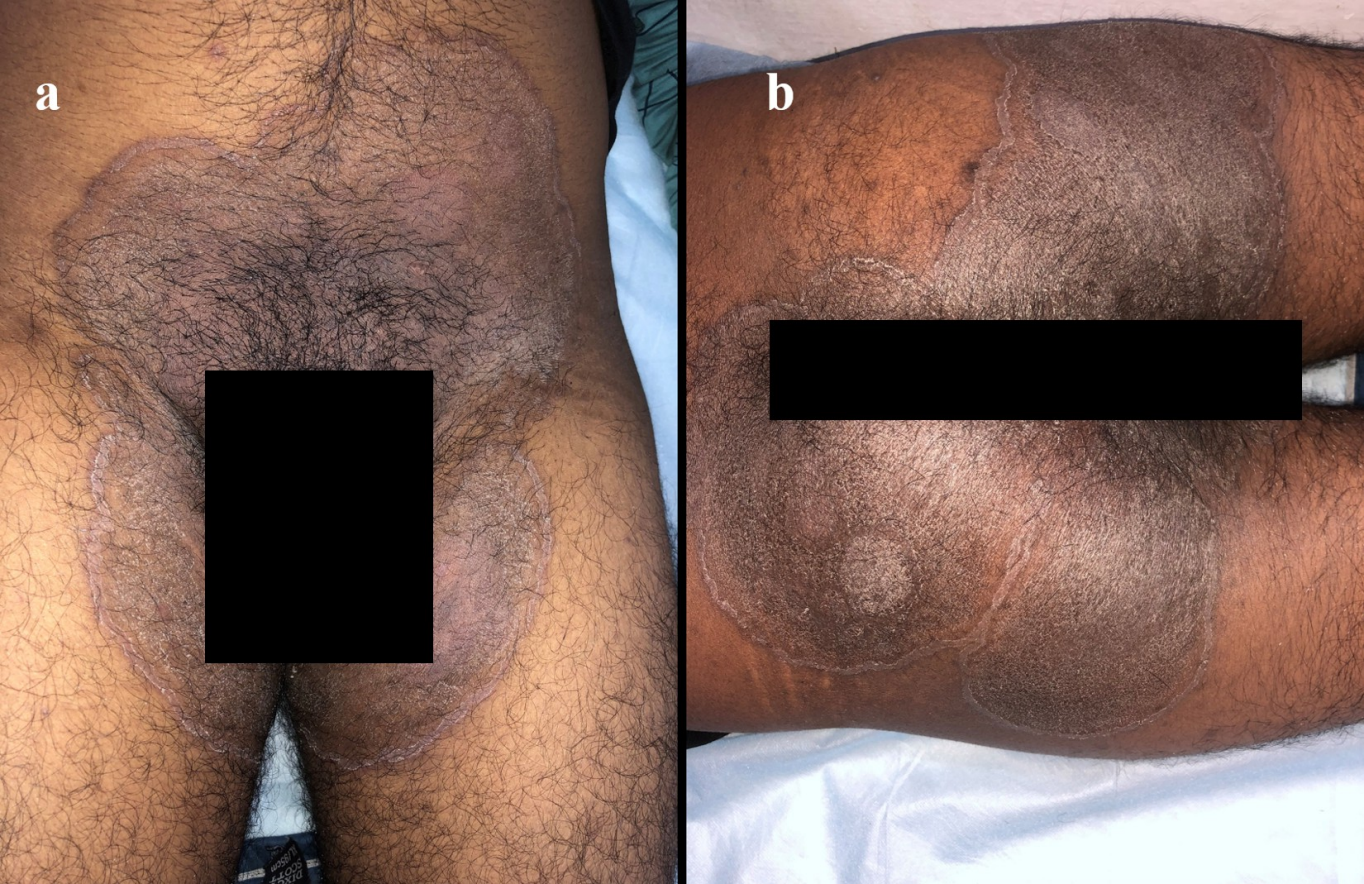
Annular plaques with raised edges and scaly central region over groins (a) and buttocks (b). Note the prominent pigmentation, a common feature seen in *T*. *indotineae* infections in Indian patients.

The ongoing outbreak of dermatophytosis in India is characterized by extensive and difficult—to-treat chronic and chronic relapsing infection of the body (tinea corporis) and the groins (tinea cruris) (Fig 1) [31]. The possible underlying factors driving the outbreak of recalcitrant infections in India are multiple but not limited to over-the-counter availability and use of combination steroid–antifungal–antibiotic creams, suboptimal and irrational regimens of prescribed antifungals, and brands with low efficacy. In 2014 to 2017, highly TRB-resistant *T*. *interdigitale* strains causing tinea cruris and tinea corporis infections were identified in North India [3,4,25]. TRB is an allylamine antifungal used orally and topically as a first-line drug in the therapy of dermatophyte infections. TRB resistance has been predominantly attributed to point alterations in the *SQLE* target gene, a key enzyme in the ergosterol biosynthetic pathway leading to single amino acid substitutions. In 2018, Singh and colleagues reported that TRB-resistant *T*. *interdigitale* isolates from cases of tinea corporis/cruris in 3 hospitals in Delhi, India exhibited elevated minimum inhibitory concentrations (MICs range 1 to ≥32 mg/L) to TRB and had single amino acid substitutions Leu393Phe or Phe397Leu in the *SQLE*. Remarkably, a considerably high TRB resistance rate of 32% was recorded using CLSI broth microdilution method [3]. Further, whole genome sequence analysis of *Trichophyton* species causing severe superficial dermatophytosis in North India confirmed a unique *Trichophyton* strain related to an early diverging clade of the *T*. *mentagrophytes/interdigitale* complex. The study pointed out that a new population of *Trichophyton* with highly related isolates (42 SNPs difference between any 2 isolates) exhibiting high rates of in vitro antifungal resistance was driving an ongoing outbreak of dermatophytosis in India [4]. Followed by this report, in 2020, Japanese investigators Kano and colleagues identified these highly TRB-resistant *T*. *interdigitale*-like strains isolated from a Nepali patient and an Indian patient with tinea corporis in Japan as a new species, i.e., *T*. *indotineae*. The rDNA ITS region sequences of their study isolates were 100% identical to TRB-resistant strains of *T*. *interdigitale*, which were isolated in Delhi, India, and harbored alterations in *SQLE*. Similar to Indian strains, the isolates exhibited high MICs (32 mg/L) for TRB and contained an amino acid substitution (Phe397Leu) in *SQLE*. To avoid confusion in the taxonomy of the *T*. *mentagrophytes/interdigitale* complex, the highly TRB-resistant Indian strains were designated as a new species independent of *T*. *interdigitale/T*. *mentagrophytes*, according to clinical and mycological features [5].

### Molecular identification of *T*. *indotineae* based on the internal transcribed spacer (ITS) region sequences

The identification of *T*. *indotineae* is challenging in the routine microbiology laboratories due to the marked morphological similarities of the species with *T*. *mentagrophytes* and *T*. *interdigitale* [5,30]. These species cannot be distinguished by phenotypic tests, although colony reverse of *T*. *indotineae* is most often pale-brown to yellow-orange in colour and isolates are less often positive in Tween-80 opacity, urea hydrolysis, and hair perforation tests than *T*. *mentagrophytes* and *T*. *interdigitale* [30]. In fact, in the last 5 years before labelling *T*. *indotineae* as a species de novo, studies based on rDNA ITS region sequencing identified Indian *Trichophyton* strains as *T*. *mentagrophytes/interdigitale*; further, Nenoff and colleagues grouped the strains as *T*. *mentagrophytes* Type VIII [3,4,6,26,32]. Currently, *T*. *indotineae* is unequivocally identified by the ITS sequences, which differ only at 2 and 3 nucleotide positions from ITS sequences of *T*. *mentagrophytes* and *T*. *interdigitale*, respectively. The BLAST searches of ITS sequences of *T*. *indotineae* on NCBI database still show ≥99% sequence similarity with *T*. *mentagrophytes*, *T*. *interdigitale*, and *T*. *indotineae*. Therefore, to obtain accurate identification (i.e., sequence similarity of 100% with *T*. *indotineae*), ITS sequences of well-defined reference strains described by Tang and colleagues [30], importantly, primary *T*. *indotineae* strains (NUBS19006 and NUBS19007), should be included in the analysis. Further, based on *HMG* gene sequences, *T*. *indotineae* can be differentiated from *T*. *interdigitale and T*. *mentagrophytes*, which differ at 4 and 1 nucleotide positions, respectively [30]. It is important to draw attention that incorrect nomenclature of several CBS reference and neotype strains of *Trichophyton* spp. in the public database results in misidentification warranting updating of the database [32].

## Global spread of terbinafine-resistant *T*. *indotineae* strains

The spread of *T*. *indotineae* in Europe was noticed as early as in 2011 in Germany and most recently in 2019 to 2020, several cases have been reported from other European countries related to travel and migration [11–17,22,23,33]. Notably, 2 cases of tinea corporis and tinea cruris due to *T*. *indotineae* reported recently from Germany occurred in 2011 and 2013, even before the outbreak of dermatophytosis in India was recognised. Both the patients had travel links to India [12]. However, the strains had low TRB MICs and no *SQLE* alterations associated with TRB resistance was observed, suggesting that the early outbreak strains were probably not resistant to TRB. These findings correlate with absence of reports of TRB resistance until 2017 in India. Another report from Germany highlights that 29 patients with *T*. *indotineae* infections occurring during 2016 to 2020 had a history of travel to India, Pakistan, Bangladesh, Iraq, Bahrain, Libya, and other unspecified countries [15]. Notably, TRB-resistant *T*. *indotineae* strains isolated from cases in Germany, Denmark, and Switzerland during 2016 to 2020 exhibited Phe397Leu and Leu393Phe amino acid substitutions that confer resistance to TRB [11,15,22,33]. Since 2018, several cases of clinically resistant tinea corporis with extensive lesions that do not respond to TRB have been reported from France [13,14]. These patients were either recent immigrants or born in a country on the Indian subcontinent. The first case series of TRB-resistant *T*. *indotineae* infection in Canadian patients also emphasise travel or immigration from northern India as the source of *T*. *indotineae* [18]. The global reports of *T*. *indotineae* are listed in Table 1, which predominantly spread from 2016 onwards from the Indian subcontinent. Interestingly, Jabet and colleagues screened rDNA ITS sequences (in GenBank) of *T*. *indotineae* through March 2021 and observed widespread dissemination of the Indian strain with 12.8% of known sequences in the GenBank were from the Middle East and 9.6% from Europe. Remarkably, 98.8% of the sequences were of human origin and 6 sequences indicated an animal origin [13].

**Table 1:** Details of *T*. *indotineae* infections outside India.

Countries reporting *T*. *indotineae*: Year of publication	Year of collection of strains	*T*. *indotineae* strains/total number of *Trichophyton* spp. strains investigated	Travel history or patients origin	Patients age group; clinical details	Treatment and outcome	TRB MICs (mg/L) / substitution in *SQLE* gene	Azole MICs (mg/L)	References
**East and South East Asia**								
Japan: 2020	2019	2/2	Nepal and India	27 and 47-years; tinea faciei/ corporis/ cruris	Oral TRB→ITC/RVZ→ITR Topical BFN, TRB, KTC, LUZ **Outcome** Complete cure with ITC and LUZ	>32 /Phe397Leu	ITC: 0.03 CTZ: 0.06–4 MCZ: 0.125–8 LUZ: <0.03 RVZ: 0.03–0.5	[5]
Vietnam: 2022	2020	1/1	Autochthonous	27 years; tinea corporis	Oral ITC Topical KTC **Outcome** Complete cure	0.25	ITC: 0.125 VRC: 0.25	[8]
Cambodia: 2019	2019	1/4	NM	26 years; tinea corporis	NM	ND	ND	[9]
**Middle East Asia**								
Iran: 2019	2016–2018*	116/1003*	Australia/ India/ Iran/Oman	NM; tinea corporis/ cruris/ faciei	NM	0.003- ≥32/ ND	ND	[20]
Iran: 2020	NM	4/4	Autochthonous	4–64 years; tinea corporis/ cruris/ pedis	Oral FLU→ prednisolone+methotrexate→ TRB→ITC→VRC/ FLU→ITC→VRC/ TRB→ITC Topical TRB, CTZ and SER. **Outcome** Complete cure with oral VRC or ITC	≥8/Phe397Leu	ITC: ≥4 FLU: ≥16 VRC: 0.2–0.5 PSZ: 0.06–0.313	[21]
Iran: 2020	2016–2018	28/141	Autochthonous	NM; tinea corporis/ cruris	NM	>32/ Phe397Leu, Leu393Ser	ITC: 0.062–2 EFN: 0.001–0.125 CTZ: 0.5–32 LUZ: 0.0004–0.015 GRE: 0.25–4 AMO: 0.125–4 CPO: 0.062–1	[19]
Iraq: 2021	2016–2021	18/48	Autochthonous	4 months-70 years; tinea corporis/ faciei/ manuum/ capitis / pedis/ cruris/ unguium/ barbae	Topical Steroids **Outcome** lesions either enlarged or flared again	ND	ND	[24]
Iran: 2022	2018–2019	10/82	Autochthonous	11–60 years; tinea corporis/ cruris/ pedis/ capitis/ faciei/ manuum	NM	0.015–32/ Phe397Leu	ITC 0.06–16 FLU 0.125–16 VRC 0.125–8 KTC: 0.125–16 PSZ: 0.125–16 AMB: 0.125–16	[7]
**Europe**								
Germany: 2020	2011–2020	5/5	Autochthonous/ India/Yemen	20–38 years; tinea corporis/ cruris/ manuum/ faciei	Oral ITC →TRB/ Several cycle of TRB and ITC Topical CTZ, CPO, TRB and MCZ **Outcome** Majority of patients experienced recurrence.	≤ 0.06	FLU: 16–64 ITC: 0.03–0.06 VRC: 0.125–0.5	[12]
Germany: 2019	2019	1/1	Bahrain	6 months; tinea corporis/ cruris	Topical TRB→ MCZ and CPO **Outcome** Complete cure with MCZ and CPO	>0.2/ Phe397Leu	ND	[10]
Germany: 2020	2016–2020	29/29	Autochthonous/ India/Pakistan/ Bangladesh/ Iraq/Bahrain/ Libya	6 months–58 years; tinea cruris/ corporis/ faciei/ manuum/ unguium/ pedis	Treatment mentioned for 4 patients only. Oral FLU and TRB→ITC Topical TRB, MCZ, CPO, CTZ, SER **Outcome** Complete cure with oral ITC	0.2-16/ Phe397Leu, Leu393Phe	ITC: 0.03–0.5 VRC: 0.03–0.5	[15]
Switzerland: 2021	2009–2019	11/162	India/Bangladesh/ Thailand	31–41 years; tinea cruris/ corporis/ faciei/ pedis	Oral TRB or ITC or FLU Topical TRB, KTC, CLT, AMO, MCZ, ECZ, ISA **Outcome** No follow up	≥4/ Phe397Leu	MIC values not mentioned	[22]
France: 2022	2017–2021	7/10	France/ India/ Bangladesh/ Myanmar	16–53 years; tinea cruris/ corporis	Oral TRB/ TRB→GRE/ TRB→GRE→ITC Topical TRB, ECZ, BFN, OMC, MCZ, CPO **Outcome** Treatment failed in patients harbouring strains of TRB MICs 2->8 mg/L	0.06 ->8/ Phe397Leu, Leu393Ser	ITC: 0.016–0.25 VRC: 0.03–0.5 AMO: 0.01–0.125	[13]
France: 2022	2018–2019	7/350	India/ Bangladesh/ Sri Lanka	20–57 years; tinea corporis/ cruris	Oral TRB/ TRB→ITC/ TRB→GRE→ITC/ FLU Topical BFN, CPO, TRB **Outcome** Cure with oral ITC and oral ITC with topical BFN	0.014-4/ Phe397Leu, Leu393Ser	ITC: 015–16 VRC: 0.125–2 PSZ 0.03–0.5 ISA: 0.125–4	[14]
Belgium: 2020	2018	1/182	Autochthonous	25 years; tinea cruris/corporis/capitis	Oral TRB Topical Sulconazole nitrate and KTC **Outcome** No follow up	4/ Phe397Leu	ITC: 0.016 VRC: 0.5 AMO: 0.06	[16]
Greece: 2019	2010–2019	9/112	Greece/Syria/Iran	9 months to 90 years; tinea cruris/corporis	Oral TRB or ITC or FLU Topical TRB and azole ointment **Outcome** Improvement with topical azoles and oral ITC	0.25-8/ Phe397Leu, Leu393Ser	ITC: 0.016–0.125 VRC: 0.03–0.5 AMO: 0.125–0.25	[17]
Switzerland, Greece, Estonia, Finland: 2021	2020	11/96	India/Bangladesh/ Pakistan/The United Arab Emirates	NM; tinea/ corporis/ capitis	Oral TRB→ ITC/ TRB→ FLU/ TRB→GRE **Outcome** NM	ND	ND	[23]
Denmark: 2022	2019–2020	7/63	Autochthonous	NM	NM	2- ≥4 Phe397Leu, Leu393Phe	ITC: ≤ 0.016–0.06 VRC: 0.06–0.5 ISA: 0.06–0.5 PSZ: 0.008–0.125 OLO: 0.008–0.03	[11]
Denmark: 2019	2013–2018	1/14	Autochthonous	25 years; tinea cruris/corporis/faciei/ pedis	Oral TRB/ TRB, ITC/ TRB, ITC, FLU/ TRB, ITC, GRE/ TRB, ITC, FLU, GRE Topical TRB, AMO, CPO, KTC and MCZ-hydrocortisone combination **Outcome** NM	≥4 Phe397Leu	ND	[33]
**North America**								
Canada: 2022	2021	8/8	India/Thailand	26–78 years; tinea cruris/corporis/faciei/ pedis	Oral TRB/ TRB→FLU/ ITC/ ITC→ FLU Topical TRB, KTC, CTZ, clobetasol, betamethasone, betamethasone dipropionate and fluocinonide **Outcome** Complete cure with oral ITC in 1 patient	ND	ND	[18]

AMO, amorolfine; AMB, Amphotericin B; BFN, bifonazole; CPO, ciclopirox olamine; CTZ, clotrimazole; ECZ, econazole; EFN, efinaconazole; FLU, fluconazole; GRE, griseofulvin; ISA, isavuconazole; ITC, itraconazole; KTC, ketoconazole; LUZ, luliconazole; MCZ, miconazole; ND, not determined; NM, not mentioned; OLO, olorofim; OMC, omoconazole; PSZ, posaconazole; RVZ, ravuconazole; SER, sertaconazole; TRB, terbinafine; VRC, voriconazole.

*Out of 1,003, 397 were strains from patient samples (collected during 2016–2018) and the remaining were ITS (internal transcribed spacer) region sequences retrieved from GenBank.

**→** denotes “followed by”.

## Developments in the antifungal susceptibility testing (AFST) and mechanism of resistance in *T*. *indotineae*

AFST of dermatophytes is not routinely performed as both the reference CLSI and EUCAST methods are time consuming, and technical constraints related to slow growth and bacterial contamination remain a challenge [34,35]. The EUCAST method recommends chloramphenicol and cycloheximide supplemented growth media (to inhibit bacterial contamination) with a spectrophotometric endpoint reading using 50% growth inhibition [34]. EUCAST tentative epidemiological cutoff values (ECOFFS) for *T*. *indotineae* successfully demarcate isolates with and without *SQLE* resistance alterations [11]. No clinical breakpoints for TRB have been established by CLSI, and several reports have adopted variable MIC values (0.25 and ≥2 mg/L) for identification of TRB-resistant isolates. [3,4,15,26]. Further, for determination of MICs against azole drugs, variable criteria have been adopted by CLSI and EUCAST. For example, in itraconazole (ITC) testing, trailing growth may impact the observed resistance rate particularly when the MICs are determined using a stringent endpoint (90% inhibition) as adopted by CLSI in comparison with the 50% endpoint adopted by EUCAST [11,34,35]. The susceptibility testing by both the reference methods warrant further harmonization and standardization.

With the increasing number of resistant and recalcitrant *T*. *indotineae* cases, understanding the mechanism of resistance remains vital. In TRB-resistant *Trichophyton* species, alterations in the *SQLE* gene leading to amino acid substitutions at one of the 4 positions (Leu^393^, Phe^397^, Phe^415^, and His^440^) have been linked to resistance [36]. The most common substitution in *T*. *indotineae* strains reported worldwide is Phe397Leu in 95% of the studies leading to high TRB MICs (range: 1 to >32 mg/L) followed by Leu393Phe (MIC range: 1 to 64 mg/L) and Leu393Ser (MIC range: 0.5 to 1 mg/L) [3–5,7,10,11,13–17,19,21,22,25–28]. Notably, Phe397Leu or Leu393Phe substitutions confer high-level TRB resistance in *T*. *mentagrophytes*, *T*. *interdigitale*, *T*. *indotineae*, and *T*. *rubrum* [37]. Yamada and colleagues introduced the 2 common abovementioned amino acid substitutions into the endogenous *SQLE* gene of a TRB-sensitive *Arthroderma vanbreuseghemii* (formerly *T*. *mentagrophytes*) strain and showed that resistance to TRB in *A*. *vanbreuseghemii* transformants was due to the respective point alterations [36]. A newly developed DermaGenius Resistance real-time PCR assay is found to be highly efficacious in differentiation of *SQLE* wild type (*T*. *indotineae* susceptible) from mutant genotypes harbouring Phe397Leu or Leu393Phe substitution. However, the significance and clinical utility of such assays in patient management needs to be investigated [38]. Importantly, azole resistance in *T*. *indotineae* has been observed in one-third of reports from India and Europe [3,4,12,14,25–27]. *T*. *indotineae* strains with the double substitutions in the *SQLE* gene, i.e., Phe397Leu and Ala448Thr, exhibit increased MIC values of fluconazole, ITC, and voriconazole (VRC) [27]. However, the speculation that these double mutants lead to FLU, ITC, and VRC resistance in *T*. *indotineae* need to be experimentally investigated. A recent study highlighted that azole resistance in *T*. *indotineae* is due to overexpression of the *TinCYP51B* gene encoding sterol 14α-demethylases enzyme [39].

## Multidrug-resistant *T*. *indotineae* and its impact on the treatment of dermatophytosis

TRB resistance rates ranging 17% to 75% and varying levels of ITC resistance up to 25% have been reported in *T*. *indotineae* strains from India [3,25,26]. In addition, several studies have reported high in vitro MIC values of VRC (range: 2 to >16 mg/L) and griseofulvin (range: 4 to 128 mg/L) [3,4,26]. It is important to emphasise that the treatment options for dermatophytosis are restricted and resistance to existing antifungals leaves no options for clinicians to treat severe persistent skin infections [40]. ITC remains the most effective antifungal for dermatophyte infections, with rising resistance to TRB. However, oral formulations of ITC have erratic absorption patterns leading to wide fluctuations in its serum concentrations. Although, monitoring of serum levels of ITC as in systemic mycoses is important for effective treatment outcome [41,42]. However, the high cost burden associated with regular therapeutic drug monitoring for this extremely prevalent infection, especially in lower-middle countries, is not practical. A single centre-based study reported that higher doses and longer durations of TRB therapy could overcome treatment failure associated with TRB-resistant strains [29]. Thus, appropriate dosage of TRB in treatment of dermatophytosis could prevent the usage of azole drugs and development of resistance against azole-based antifungal drugs. Recently, in vitro synergistic interactions with varying combinations of ITC, TRB, ketoconazole, and luliconazole (LUZ) have been observed in TRB-resistant Indian strains [43,44]. However, effectiveness of TRB and ITC in combination for treatment of tinea infections in a recent randomised trial showed no added beneficial effect over treatment with ITC alone [45]. Thus, combination treatment consisting of 2 systemic antifungals has no proven clinical benefit and must be avoided as it not only adds to cost of treatment, but also exposes patients to a greater array of adverse effects. It is more useful and rationale instead to combine oral drugs with topical antifungals with high susceptibility against the prevalent strain (e.g., LUZ) especially as topical antifungals achieve much higher levels in the skin. However, extensive skin involvement, as often seen with *T*. *indotineae*, makes the option economically unfeasible in many patients. Therefore, it’s important to develop newer highly potent antifungals for oral use and also gear up the clinical application of drugs like olorofim, which have high potent in vitro activity against *T*. *indotineae* [3,4,46,47]. Finally, *T*. *indotineae* has become widespread due to travel, immigration, and subsequent local transmission in the countries warranting urgent collective efforts at the global level to prevent its further dissemination.

## References

[1] Ameen M. Epidemiology of superficial fungal infections. Clin Dermatol. 2010;28:197–201. doi: 10.1016/j.clindermatol.2009.12.005 20347663

[2] Benedict K, Jackson BR, Chiller T, Beer KD. Estimation of direct healthcare costs of fungal diseases in the United States. Clin Infect Dis. 2019;68:1791–1797. doi: 10.1093/cid/ciy776 30204844 PMC6409199

[3] Singh A, Masih A, Khurana A, Singh PK, Gupta M, Hagen F, et al. High terbinafine resistance in *Trichophyton interdigitale* isolates in Delhi, India harbouring mutations in the squalene epoxidase gene. Mycoses. 2018;61:477–484. 29577447 10.1111/myc.12772

[4] Singh A, Masih A, Monroy-Nieto J, Singh PK, Bowers J, Travis J, et al. A unique multidrug-resistant clonal *Trichophyton* population distinct from *Trichophyton mentagrophytes/Trichophyton interdigitale* complex causing an ongoing alarming dermatophytosis outbreak in India: Genomic insights and resistance profile. Fungal Genet Biol. 2019;133:103266. 31491507 10.1016/j.fgb.2019.103266

[5] Kano R, Kimura U, Kakurai M, Hiruma J, Kamata H, Suga Y, et al. *Trichophyton indotineae* sp. nov.: A new highly terbinafine-resistant anthropophilic dermatophyte species. Mycopathologia. 2020;185:947–958. 32449054 10.1007/s11046-020-00455-8

[6] Nenoff P, Verma SB, Vasani R, Burmester A, Hipler UC, Wittig F, et al. The current Indian epidemic of superficial dermatophytosis due to *Trichophyton mentagrophytes*-A molecular study. Mycoses. 2019;62:336–356. 30561859 10.1111/myc.12878

[7] Pashootan N, Shams-Ghahfarokhi M, Chaichi Nusrati A, Salehi Z, Asmar M, Razzaghi-Abyaneh M. Phylogeny, antifungal susceptibility, and point mutations of *SQLE* gene in major pathogenic dermatophytes isolated from clinical dermatophytosis. Front Cell Infect Microbiol. 2022;12:851769. 35372131 10.3389/fcimb.2022.851769 PMC8972121

[8] Ngo TMC, Ton Nu PA, Le CC, Ha TNT, Do TBT, Tran TG. First detection of *Trichophyton indotineae* causing tinea corporis in central Vietnam. Med Mycol Case Rep. 2022;36:37–41. 35620657 10.1016/j.mmcr.2022.05.004 PMC9127533

[9] Uhrlass S, Sithach M, Koch D, Wittig F, Muetze H, Krueger CNP. *Trichophyton mentagrophytes*—A new genotype in Cambodia. J. Fungi. 2019;5:460.

[10] Süß A, Uhrlaß S, Ludes A, Verma SB, Monod M, Krüger C, et al. Ausgeprägte tinea corporis durch ein terbinafin resistentes *Trichophyton-mentagrophytes*-isolat vom indischen genotyp bei einem säugling aus Bahrain in Deutschland. Der Hautarzt. 2019;70:888–896. 10.1007/s00105-019-4431-7 31098692

[11] Astvad KMT, Hare RK, Jørgensen KM, Saunte DML, Thomsen PK, et al. Increasing terbinafine resistance in Danish *Trichophyton* isolates 2019–2020. J Fungi (Basel). 2022;8:150. doi: 10.3390/jof8020150 35205904 PMC8879722

[12] Brasch J, Gräser Y, Beck-Jendroscheck V, Voss K, Torz K, Walther G, et al. "Indian" strains of *Trichophyton mentagrophytes* with reduced itraconazole susceptibility in Germany. J Dtsch Dermatol Ges. 2021;19:1723–1727. 10.1111/ddg.14626 34850554

[13] Jabet A, Brun S, Normand AC, Imbert S, Akhoundi M, Dannaoui E, et al. Extensive dermatophytosis caused by terbinafine-resistant *Trichophyton indotineae*, France, Emerg Infect Dis. 2022;28:229–233. 34932456 10.3201/eid2801.210883 PMC8714191

[14] Dellière S, Joannard B, Benderdouche M, Mingui A, Gits-Muselli M, Hamane S, et al. Emergence of difficult-to-treat tinea corporis caused by *Trichophyton mentagrophytes* complex isolates, Paris. France. Emerg Infect Dis. 2022;28:224–228. 34932462 10.3201/eid2801.210810 PMC8714205

[15] Nenoff P, Verma SB, Ebert A, Süß A, Fischer E, Auerswald E, et al. Spread of terbinafine-resistant *Trichophyton mentagrophytes* type VIII (India) in Germany-"the tip of the iceberg?". J Fungi (Basel). 2020;6:207. 10.3390/jof6040207 PMC7712673 33027904

[16] Sacheli R, Harag S, Dehavay F, Evrard S, Rousseaux D, Adjetey A, et al. Belgian national survey on tinea capitis: epidemiological considerations and highlight of terbinafine-resistant *T*. *mentagrophytes* with a mutation on *SQLE* gene. J Fungi (Basel). 2020;6:195. 10.3390/jof6040195 PMC7712443 33003309

[17] Siopi M, Efstathiou I, Theodoropoulos K, Pournaras S, Meletiadis J. Molecular epidemiology and antifungal susceptibility of *Trichophyton* isolates in Greece: emergence of terbinafine-resistant *Trichophyton mentagrophytes* type VIII locally and globally. J Fungi (Basel). 2021;7:419. 34072049 10.3390/jof7060419 PMC8229535

[18] Posso-De Los Rios CJ, Tadros E, Summerbell RC, Scott JA. Terbinafine resistant *Trichophyton indotineae* isolated in patients with superficial dermatophyte infection in Canadian patients. J Cutan Med Surg. 2022;12034754221077891. doi: 10.1177/12034754221077891 35144480

[19] Taghipour S, Shamsizadeh F, Pchelin IM, Rezaei-Matehhkolaei A, Zarei Mahmoudabadi A, Valadan R, et al. Emergence of terbinafine resistant *Trichophyton mentagrophytes* in Iran, harboring mutations in the squalene epoxidase (SQLE) gene. Infect Drug Resist. 2020;13:845–850. 32214830 10.2147/IDR.S246025 PMC7078656

[20] Taghipour S, Pchelin IM, Zarei Mahmoudabadi A, Ansari S, Katiraee F, Rafiei A, et al. *Trichophyton mentagrophytes* and *T interdigitale* genotypes are associated with particular geographic areas and clinical manifestations. Mycoses. 2019;62:1084–1091. 10.1111/myc.12993 31444823

[21] Fattahi A, Shirvani F, Ayatollahi A, Rezaei-Matehkolaei A, Badali H, Lotfali E, et al. Multidrug-resistant *Trichophyton mentagrophytes* genotype VIII in an Iranian family with generalized dermatophytosis: report of four cases and review of literature. Int J Dermatol. 2021;60:686–692. 33047849 10.1111/ijd.15226

[22] Klinger M, Theiler M, Bosshard PP. Epidemiological and clinical aspects of *Trichophyton mentagrophytes/Trichophyton interdigitale* infections in the Zurich area: a retrospective study using genotyping. J Eur Acad Dermatol Venereol. 2021;35:1017–1025. 33411941 10.1111/jdv.17106

[23] Saunte DML, Pereiro-Ferreirós M, Rodríguez-Cerdeira C, Sergeev AY, Arabatzis M, Prohić A, et al. Emerging antifungal treatment failure of dermatophytosis in Europe: take care or it may become endemic. J Eur Acad Dermatol Venereol. 2021;35:1582–1586. doi: 10.1111/jdv.17241 33768571

[24] Sharquie KE, Jabbar RI. Major Outbreak of dermatophyte infections leading into imitation of different skin diseases: *Trichophyton mentagrophytes* is the main criminal fungus. J Turk Acad Dermatol. 2021;15:91–100.

[25] Rudramurthy SM, Shankarnarayan SA, Dogra S, Shaw D, Mushtaq K, Paul RA, et al. Mutation in the squalene epoxidase gene of *Trichophyton interdigitale* and *Trichophyton rubrum* associated with allylamine resistance. Antimicrob Agents Chemother. 2018;62:e02522–e02517. 29530857 10.1128/AAC.02522-17 PMC5923174

[26] Ebert A, Monod M, Salamin K, Burmester A, Uhrlaß S, Wiegand C, et al. Alarming India-wide phenomenon of antifungal resistance in dermatophytes: A multicentre study. Mycoses. 2020;63:717–728. doi: 10.1111/myc.13091 32301159

[27] Kong X, Tang C, Singh A, Ahmed SA, Al-Hatmi AMS, Chowdhary A, et al. Antifungal susceptibility and mutations in the squalene epoxidase gene in dermatophytes of the *Trichophyton mentagrophytes* species complex. Antimicrob Agents Chemother. 2021;65:e0005621. 33972254 10.1128/AAC.00056-21 PMC8284460

[28] Burmester A, Hipler UC, Uhrlaß S, Nenoff P, Singal A, Verma SB, et al. Indian *Trichophyton mentagrophytes* squalene epoxidase erg1 double mutants show high proportion of combined fluconazole and terbinafine resistance. Mycoses. 2020 Jul 29. doi: 10.1111/myc.13150 Epub ahead of print . 32725892

[29] Khurana A, Masih A, Chowdhary A, Sardana K, Borker S, Gupta A, et al. Correlation of *in vitro* susceptibility based on MICs and *SQLE* mutations with clinical response to terbinafine in patients with tinea corporis/cruris. Antimicrob Agents Chemother. 2018. pii: AAC.01038-18. 10.1128/AAC.01038-18 PMC6256768 30275090

[30] Tang C, Kong X, Ahmed SA, Thakur R, Chowdhary A, Nenoff P, et al. Taxonomy of the *Trichophyton mentagrophytes/T*. *interdigitale* species complex harboring the highly virulent, multiresistant genotype T. *indotineae*. Mycopathologia. 2021;186:315–326. 33847867 10.1007/s11046-021-00544-2 PMC8249266

[31] Verma SB, Panda S, Nenoff P, Singal A, Rudramurthy SM, Uhrlass S, et al. The unprecedented epidemic-like scenario of dermatophytosis in India: Epidemiology, risk factors and clinical features. Indian J Dermatol Venereol Leprol. 2021;87:154–175. 33769736 10.25259/IJDVL_301_20

[32] Chowdhary A, Singh A, Singh PK, Khurana A, Meis JF. Perspectives on misidentification of *Trichophyton interdigitale/Trichophyton mentagrophytes* using internal transcribed spacer region sequencing: Urgent need to update the sequence database. Mycoses. 2019;62:11–15. 30367553 10.1111/myc.12865

[33] Saunte DML, Hare RK, Jørgensen KM, Jørgensen R, Deleuran M, Zachariae CO, et al. Emerging terbinafine resistance in *Trichophyton*: Clinical characteristics, squalene epoxidase gene mutations, and a reliable EUCAST method for detection. Antimicrob Agents Chemother. 2019;63:e01126–e01119. 31383665 10.1128/AAC.01126-19 PMC6761549

[34] The European Committee on Antimicrobial Susceptibility Testing. Overview of antifungal ECOFFs and clinical breakpoints for yeasts, moulds and dermatophytes using the EUCAST E.Def 7.3, E.Def 9.4 and E.Def 11.0 procedures. Version 3, 2022. http://www.eucast.org."

[35] CLSI. Reference Method for Broth Dilution Antifungal Susceptibility Testing of Filamentous Fungi. 3rd ed. CLSI standard M38. Wayne, PA: Clinical and Laboratory Standards Institute; 2017.

[36] Yamada T, Maeda M, Alshahni MM, Tanaka R, Yaguchi T, Bontems O, et al. Terbinafine resistance of *Trichophyton* clinical isolates caused by specific point mutations in the squalene epoxidase gene. Antimicrob Agents Chemother. 2017;61:e00115–e00117. 10.1128/AAC.00115-17 PMC5487658 28416557

[37] Rogers TR, Verweij PE, Castanheira M, Dannaoui E, White PL, Arendrup MC; Subcommittee on Antifungal Susceptibility Testing (AFST) of the ESCMID European Committee for Antimicrobial Susceptibility Testing (EUCAST). Molecular mechanisms of acquired antifungal drug resistance in principal fungal pathogens and EUCAST guidance for their laboratory detection and clinical implications. J Antimicrob Chemother. 2022; dkac161. [Epub ahead of print. doi: 10.1093/jac/dkac161 ]. 35703391 PMC9333407

[38] Singh A, Singh P, Dingemans G, Meis JF, Chowdhary A. Evaluation of DermaGenius resistance real-time polymerase chain reaction for rapid detection of terbinafine-resistant *Trichophyton* species. Mycoses. 2021;64:721–726. 33760310 10.1111/myc.13271

[39] Yamada T, Yaguchi T, Maeda M, Alshahni MM, Salamin K, Guenova E, et al. Gene amplification of *CYP51B*: a new mechanism of resistance to azole compounds in *Trichophyton indotineae*. Antimicrob Agents Chemother. 2022:e0005922. doi: 10.1128/aac.00059-22 35546111 PMC9211412

[40] Khurana A, Sardana K, Chowdhary A. Antifungal resistance in dermatophytes: Recent trends and therapeutic implications. Fungal Genet Biol. 2019;132:103255. doi: 10.1016/j.fgb.2019.103255 31330295

[41] Singh S, Chandra U, Anchan VN, Verma P, Tilak R. Limited effectiveness of four oral antifungal drugs (fluconazole, griseofulvin, itraconazole and terbinafine) in the current epidemic of altered dermatophytosis in India: results of a randomized pragmatic trial. Br J Dermatol. 2020;183:840–846. doi: 10.1111/bjd.19146 32538466

[42] Khurana A, Agarwal A, Singh A, Sardana K, Ghadlinge M, Agrawal D, et al. Predicting a therapeutic cut-off serum level of itraconazole in recalcitrant tinea corporis and cruris- A prospective trial. Mycoses. 2021;64:1480–1488. doi: 10.1111/myc.13367 34532888

[43] Bidaud AL, Schwarz P, Chowdhary A, Dannaoui E. *In Vitro* antifungal combination of terbinafine with itraconazole against isolates of *Trichophyton* species. Antimicrob Agents Chemother. 2022;66:e0144921. 34633845 10.1128/AAC.01449-21 PMC8765265

[44] Sardana K, Gupta A, Sadhasivam S, Gautam RK, Khurana A, Saini S, et al. Checkerboard analysis to evaluate synergistic combinations of existing antifungal drugs and propylene glycol monocaprylate in isolates from recalcitrant tinea corporis and cruris patients harboring squalene epoxidase gene mutation. Antimicrob Agents Chemother. 2021;65:e0032121. doi: 10.1128/AAC.00321-21 34097482 PMC8284445

[45] Singh SK, Subba N, Tilak R. Efficacy of terbinafine and itraconazole in different doses and in combination in the treatment of tinea infection: a randomized controlled parallel group open labeled trial with clinico-mycological correlation. Indian J Dermatol. 2020;65:284–289. doi: 10.4103/ijd.IJD_548_19 32831369 PMC7423219

[46] Singh A, Singh P, Meis JF, Chowdhary A. *In vitro* activity of the novel antifungal olorofim against dermatophytes and opportunistic moulds including *Penicillium* and *Talaromyces* species. J Antimicrob Chemother. 2021;76:1229–1233. 33421073 10.1093/jac/dkaa562 PMC8050765

[47] Astvad KMT, Jørgensen KM, Hare RK, Datcu R, Arendrup MC. Olorofim susceptibility testing of 1,423 Danish mold isolates obtained in 2018–2019 confirms uniform and broad-spectrum activity. Antimicrob Agents Chemother. 2020;65:e01527–e01520. doi: 10.1128/AAC.01527-20 33020160 PMC7927864

